# Evaluation of the Diagnostic Accuracy of Exhaled Nitric Oxide as a Marker of Infection and Sepsis in Emergency Department Patients

**DOI:** 10.1155/emmi/8911242

**Published:** 2025-03-11

**Authors:** Kendal Farrar, Jacob L. Haapala, Kirsten A. Dalrymple, Lauren R. O'Keefe, Carter R. Anderson, Russ L. Morris, Michael D. Zwank

**Affiliations:** ^1^Emergency Department, Methodist Hospital, St. Louis Park, Minnesota, USA; ^2^Biostatistics Department, HealthPartners Institute, Bloomington, Minnesota, USA; ^3^Research & Development Department, Vail Scientific, Bloomington, Minnesota, USA; ^4^Emergency Department, Regions Hospital, St. Paul, Minnesota, USA

## Abstract

**Background:** Early identification of septic patients in the ED is important, but high patient volumes and lengthy wait times often delay workups, and typically used noninvasive triage screening tools such as vital signs and qSOFA have poor sensitivity. Nitric oxide (NO) is a molecule in the blood that has been found to be upregulated in sepsis. Since it has a very short half-life in blood, its measurement can be challenging. We aimed to determine if exhaled NO could be used to help predict bacterial infection and sepsis.

**Methods:** Emergency department patients with concern for infection were assessed for enrollment. Patients were included if blood cultures were ordered by the ED provider. The exhaled breath NO levels of enrolled subjects were measured. A score (vital signs and nitric oxide [VSNO]) was then created that included triage vital signs and NO level.

**Results:** 104 patients (41 female) were enrolled. The median exhaled NO level was 9.8 parts per billion (ppb) (IQR: 5.6–17.0). Sixty-two (60%) patients were diagnosed with bacterial infection, and of those, 54 (52%) patients were diagnosed with sepsis. Using cut points of < 7 or > 12 ppb, the VSNO score demonstrated a sensitivity of 0.89 (95% CI: 0.77–0.96) and a specificity of 0.48 (95% CI: 0.34–0.63) for predicting sepsis. The score showed a sensitivity of 0.82 (95% CI: 0.70–0.91) and a specificity of 0.45 (95% CI: 0.30–0.64) for predicting bacterial infection.

**Conclusions:** Exhaled NO measurement combined with vital signs has a high sensitivity for the detection of bacterial infection and sepsis. In a clinical setting, this score would be immediately available at the point of patient triage and would help to direct downstream evaluation and care. Further research is warranted.

## 1. Introduction

Identification of sepsis is both critical and challenging. Mortality rates of 24%–32% have been described and mortality increases by 7.6% for every hour of antibiotic administration is delayed [1, 2]. This is particularly important in septic shock; the Surviving Sepsis Campaign International Guidelines recommends that administration of IV antimicrobials should be initiated within 3 hours of recognition of sepsis, and within 1 hour for septic shock [3]. Identifying potentially septic emergency department (ED) patients at the time of triaging or in a prehospital setting such as an ambulance or urgent care would allow for more appropriate downstream evaluation and care. The ability to better triage these patients is especially important given the multihour wait times prevalent in overcrowded EDs nationwide. Standard screening (two or more SIRS vital signs outside of normal range plus the suspicion of infection) demonstrates a sensitivity of only 40% [4]. The quick sequential organ failure assessment (qSOFA) score demonstrates a high specificity (82%) but likewise has a low sensitivity (46%), which again limits its utility as a triage screening tool [5, 6].

Nitric oxide (NO) is a signaling molecule which plays an important signaling role in vasodilation, neurotransmission, cardiac function, and immune system activation and is known to be dysregulated in sepsis [7–10]. NO production affects micro- and macrocirculation differently, leading to an overall mixed clinical effect. NO in microcirculation may improve capillary exchange and prevent organ dysfunction, while NO in macrocirculation causes myocardial depression, a drop in systemic vascular resistance, hypotension, and shock [11].

Previous research has found that NO production in sepsis is biphasic, although the timing and amplitude of NO production have not been entirely elucidated. In early sepsis, NO levels rise. In a model of sepsis in healthy volunteers, exhaled NO increased within 2 hours after endotoxin infusion [12]. This may represent the first “slow the infection” stage of sepsis progression and has also been seen in animal trials [13]. As sepsis progresses, however, NO levels drop, perhaps evolving into a “slow the inflammation” phase [14]. Previous research in both humans and animals suggests that this drop is associated with increased mortality [15–17].

While blood levels of NO cannot be feasibly measured due to the short half-life of the NO molecule, NO can be reliably measured in exhaled breath [18]. This application of NO measurement is already in use in asthmatic patients, where increased exhaled NO levels indicate increased inflammation of the airways. Reference ranges for fractional exhalation of NO (FeNO) in healthy adults have already been characterized in previous research [19–22]. In a study of 2200 subjects, NO levels had a median value of 16 parts per billion (ppb), with a range of 2.4–199 ppb [22]. NO values are affected by the measurement technique, exhalation rate, age, height, smoking, anti-inflammatory medications, and stress [23–25].

We hypothesized that in a cohort of ED patients, exhaled levels of NO, alone or in combination with vital signs, can be used as a marker for the detection of infection and sepsis in patients with suspicion of infection.

## 2. Methods

### 2.1. Study Setting and Population

This project was an IRB-approved prospective diagnostic accuracy study of ED patients with possible infection. The study was conducted in the EDs of two urban tertiary care hospitals within one large healthcare network. One hospital has 509 inpatient beds and an ED census of 90,000 while the other hospital has 426 inpatient beds and an ED census of 60,000. Inclusion criteria were as follows: ED patients age 18 years or older, blood cultures ordered, English speaking, ability to consent, breathing room air or oxygen by nasal cannula, ability to complete at least one exhalation breath test, and ability to have breath testing within six hours of ED arrival. Exclusion criteria were as follows: history of lung disease (asthma, COPD, or pulmonary fibrosis), pregnancy, current use of home oxygen, use of inhaled albuterol or corticosteroids within the past month, use of antibiotics at the time of presentation, and/or requirement for BiPAP or oxygen by facemask. Patients were enrolled when research assistants were available to consent patients and collect data (24/7 at the larger hospital and 9 a.m.–5 p.m. at the smaller). Data were collected from April 2022 through March 2023. FeNO (hereafter reported as NO in units of ppb) was measured in the ED and all clinical data were collected from the electronic medical record (EMR). Patients were followed until 48 h after hospital discharge.

### 2.2. Study Protocol

ED patients were screened and identified by research assistants who were monitoring the ED track board for patients who had orders placed for blood cultures. These orders could be placed while the patient was in triage by a provider who was screening the track board or after a provider had seen the patient in a patient treatment room. The research assistants were not blinded to the study aims but were unaware of patient diagnosis at the time of enrollment. Written informed consent was obtained. To collect NO concentration, each study subject exhaled through the device (EcoMedics CLD 88sp Nitric Oxide Analyzer, Eco Medics AG, Duernten Switzerland), with each result being recorded separately. One or two exhaled breaths were collected with the patient attempting to maintain a steady exhalation flow rate (FR) of 50 mL/sec for 6–8 s with the device giving visual feedback on the FR. If the subject could not successfully exhale at a 50 mL/sec steady FR, they were instructed to exhale up to two times into the device at a harder FR. In either case, if more than one breath measurement was successful, the measurements were averaged. If the subject could not complete this task they were excluded from the study. NO levels were adjusted using a previously described calculation to account for the patient's actual FR [26]. The expression used for the correction was

(1) $$\begin{array}{c} {\text{NO}}_{\text{corrected}}=\frac{{\text{NO}}_{\text{at⁣measured⁣FR}}*20}{\left(208.6795*{\text{FR}}^{-0.5995}\right)}. \end{array}$$

All NO breathalyzer data were collected within 6 h of arrival to the ED. Clinical and laboratory data were collected from subjects' electronic charts from the window of time from when the patient arrived in the ED up until 6 h after the NO breathalyzer data were obtained, inclusive of their time in the ED and in the hospital if admitted. As the study sought to determine whether NO data correlate with the presence of sepsis, clinical data collection was thus limited to the hours surrounding the breath test. Additional data regarding the source of infection (i.e., blood and urine cultures, swab results, and relevant imaging results) were collected from the first 48 h of the patients' hospital stay. This was utilized to accurately assess the source of infection present at the time of arrival. Data were managed using the REDCap electronic data capture tool (Appendix 1). All exhaled NO data collection and all NO calculations were conducted by individuals who were unaware of patient's infection status.

A score (vital signs and nitric oxide [VSNO] - Table 1) was created for each patient utilizing their triage vital signs and their exhaled NO level. One point was given each for a RR, HR, or temperature outside of the normal ranges (similar to SIRS criteria), and one point was given for a NO value outside of optimized cut points. The optimized cutoff points were determined by maximizing Youden's J Index and prioritizing a higher sensitivity. The cut points for abnormal levels of NO were less than 7 ppb or greater than 12 ppb. A score of two or more was considered a positive VSNO score (similar to the SIRS score) (See Table 1).

### 2.3. Sepsis Diagnosis and Identification

Following patient discharge from the hospital, two physician investigators independently reviewed the patient chart to determine whether the patient had a bacterial or viral infection (including the source of infection when available), sepsis, or septic shock within 6 h of NO breathalyzer testing. The investigators were blinded to the exhaled NO levels at the time of infection and sepsis determination. This determination was made utilizing the collected lab and patient data as well as chart notes and followed Sepsis-2 guidelines (the presence of SIRS plus evidence of infection).

While the initial intention of the study was to use Sepsis-3 diagnostic criteria to determine sepsis, it became clear that these criteria were too limiting. Very few patients met Sepsis-3 criteria. There were several reasons for this: patients with a decreased GCS, patients with unstable hemodynamics, and patients requiring more than oxygen by nasal cannula were excluded. In addition, several SOFA data points (e.g., bilirubin and urine output) were infrequently obtained by ED clinicians. Functionally, this meant that of the included patient population, only patients with new thrombocytopenia and a rise in their baseline creatinine would be identified as septic. This lack of utility for ED patients has previously been a criticism of the Sepsis-3 criteria [27]. Given this, we used Sepsis-2 criteria as our study endpoint. In the current clinical practice, these are the criteria that most providers continue to use for diagnosis and charting.

### 2.4. Key Outcome Measures

The goal of the study was to investigate whether the measurement of exhaled NO, either alone or in combination with other clinical measurements, could

1. Distinguish between subjects who were and were not diagnosed with sepsis (primary aim)
2. Distinguish between subjects who were and were not diagnosed with bacterial infection (secondary aim)

### 2.5. Data Analysis

Demographics and clinical characteristics of the study sample were described using appropriate summary statistics (e.g., mean, standard deviation, median, range, and frequencies). Analyses included comparing sensitivity and specificity for classifiers (NO alone, SIRS, and VSNO) to predict disease (infection and sepsis). All statistical analyses were performed in SAS (Version 9: SAS, North Carolina, United States of America). Based on prior hospital data, we anticipated a sepsis rate of 25% among those patients for whom blood cultures are ordered. We calculated that a total enrollment of 160 patients, with 49 subjects anticipated to develop sepsis and 111 without sepsis, would produce an AUC of 0.80 with a two-sided 95% confidence interval (0.719 and 0.881).

## 3. Results

104 (41 female) patients were enrolled during a 1 year enrollment period; 83 were enrolled from Regions Hospital (24 h enrollment) and 21 from Methodist Hospital (daytime enrollment only). Enrollment was stopped after 1 year due to slower than expected patient enrollment when an interim analysis showed that enough patients had been enrolled to demonstrate clinically relevant results. Patient characteristics are summarized in Table 2.

Triage vital signs included a mean temperature of 37.4 (SD: 1.0), mean heart rate of 102 (SD: 18), mean systolic blood pressure of 129 (SD: 23), and a mean respiratory rate of 18.7 (SD: 3.2). The median exhaled NO level was 9.8 ppb (IQR: 5.6–17.0). Fifty (48%) enrolled patients were able to complete the breathalyzer at the goal rate of 50 mL/sec within 3 attempts; many elderly patients had difficulty controlling their breath at this slow steady rate. The remaining patients' NO levels were calculated from the patients' natural, generally higher exhaled FR using the aforementioned adjustment equation. A total of 4 patients provided one breath sample, 91 patients provided two breath samples, and 9 patients provided three breath samples. Of the 104 patients, 62 subjects were diagnosed with bacterial infection and of those 54 were diagnosed with sepsis (Table 3). There were no deaths. There were no cases of disagreement between the investigators in the diagnosis of bacterial infection or sepsis.

### 3.1. Triage Vital Signs and SIRS for the Detection of Sepsis and Bacterial Infection

Meeting SIRS criteria using the initial triage vital signs alone (without the addition of WBC) had a sensitivity of 0.54 (95% CI: 0.40–0.67) for the detection of sepsis, with a specificity of 0.80 (95% CI: 0.66–0.90) (Table 4). The triage vital signs produced a sensitivity of 0.40 (95% CI: 0.28–0.54) and a specificity of 0.67 (95% CI: 0.50–0.80) for detecting bacterial infection.

Meeting SIRS criteria using the initial triage vital signs including the WBC (once available) had a sensitivity of 0.83 (95% CI: 0.71–0.92) for the detection of sepsis, with a specificity of 0.70 (95% CI: 0.55–0.82). For the detection of bacterial infection, SIRS criteria from initial vitals had a sensitivity of 0.66 (95% CI: 0.53–0.78) and a specificity of 0.55 (95% CI: 0.39–0.70).

### 3.2. Nitric Oxide Levels for the Detection of Sepsis

Nitric oxide levels of < 7 or > 12 ppb alone (without the addition of vital signs) demonstrated a sensitivity of 0.81 (95% CI: 0.69–0.91) and a specificity of 0.32 (95% CI: 0.20–0.47) for predicting sepsis. The PPV was 0.56 (95% CI: 0.45–0.68) with a NPV of 0.62 (95% CI: 0.41–0.80).

### 3.3. VSNO Score for the Detection of Sepsis

The addition of triage vital signs to the nitric oxide level (to create the VSNO score, as described above) improved the performance for the detection of sepsis. A score of 2+ of the VSNO criteria demonstrated a sensitivity of 0.89 (95% CI: 0.77–0.96) and a specificity of 0.48 (95% CI: 0.34–0.63) for predicting sepsis. The AUC was 0.68 (95% CI: 0.60–0.77). The PPV was 0.65 (95% CI: 0.53–0.76) with a NPV of 0.80 (95% CI: 0.61–0.92).

### 3.4. Nitric Oxide Level for the Detection of Bacterial Infection

Nitric oxide levels of < 7 or > 12 ppb demonstrated a sensitivity of 0.82 (95% CI: 0.70–0.91) and a specificity of 0.45 (95% CI: 0.30–0.61) for predicting bacterial infection. The PPV was 0.69 (95% CI: 0.57–0.79) with a NPV of 0.63 (95% CI: 0.44–0.80).

### 3.5. VSNO Score for the Detection of Bacterial Infection

A score of 2+ on the VSNO score showed a sensitivity of 0.82 (95% CI: 0.70–0.91) and a specificity of 0.45 (95% CI: 0.30–0.61) for predicting bacterial infection. The AUC was 0.64 (95% CI: 0.55–0.73). The PPV was 0.69 (95% CI: 0.57–0.79) and the NPV was 0.63 (95% CI: 0.44–0.80) -Table 5.

## 4. Discussion

Early diagnosis of bacterial infection and sepsis is paramount, but expedient identification of these patients is challenging in overcrowded EDs with lengthy wait times. Here, a point-of-care NO breathalyzer test in conjunction with vital signs was 82% sensitive for bacterial infection, and 89% sensitive for sepsis, significantly aiding in the triage of patients. While high-sensitivity testing is generally thought of as useful for ruling out disease processes, the reality is more nuanced. In the ED with high-mortality disease processes such as sepsis, an important need is to have a test that does not inappropriately lead to a patient with a cannot-miss diagnosis being sent home. As such, a test with high sensitivity is used as a screen to “catch” patients who need further testing. Other tests in the ED used in a similar manner are troponin and D-dimer, both of which have exceedingly high sensitivity but distinctly lower specificity. Relative to the sensitivity, the lower specificity of the VSNO score does indicate that there will be a number of false positive results. However, for sepsis, we speculate that most healthcare systems would accept the possible increased testing that would result from the use of the score.

Compared to vital signs alone (typically the only objective data points available during triage), the addition of exhaled NO levels improved the sensitivity of sepsis detection from 54% to 89%. The high sensitivity of the VSNO score as contrasted with vital signs alone is likely attributable to the fact that many septic patients do not present with abnormal HR, RR, or temperature. Many EDs have protocols to obtain blood work shortly after patient arrival prior to rooming, however, compared to the full SIRS criteria including the white count, the sensitivity of VSNO remains higher, with the SIRS criteria demonstrating a sensitivity of 83% in our populations.

The specificity of the VSNO score for detecting bacterial infection (0.45) and for detecting sepsis (0.48) was lower than the SIRS criteria (0.55 and 0.70, respectively), indicating that the VSNO score is best suited for ruling out sepsis among ED all-comers. This may be of utility for patients in whom high fevers and tachycardia are common on arrival but who are not septic (e.g., influenza, COVID-19, and strep throat) or in patients with conditions that mimic sepsis (such as stimulant abuse, alcohol withdrawal, pancreatitis, and acute pain). In contrast to other biomarkers such as WBC, CRP, and procalcitonin, the NO level can quickly be obtained noninvasively.

How an ED or healthcare system might utilize this test is something that can be evaluated in further research. While the sensitivity is not at a level where the VSNO score could be used alone to rule out patients, it outperforms other currently available tools. The score could be included in an algorithm/protocol that also considers patient-specific characteristics (e.g., comorbidities, physical exam findings, and duration of symptoms). Alternatively, it could just be incorporated into the context of a given patient, aiding physician gestalt in determining further patient care.

The use of the NO level alone was not as sensitive for the detection of sepsis or bacterial infection as VSNO, although it may have utility in the context of a patient's entire clinical presentation. Further work could help to define the variations in NO levels in specific types of infection, such as pneumonia.

## 5. Limitations

There were several limitations of this study. NO levels are known to be abnormal in patients with lung disease such as asthma and COPD and we excluded this potentially large group of patients from enrollment. It is certainly possible that exhaled NO levels in these patients could prove useful and this could be an area of future research. Further, NO levels are known to be affected by other biological variables such as age, atopy, smoking, certain medications, and diet, which may confound results.

Viral (*n* = 7) and bacterial (*n* = 47) sepsis were combined in the analysis for sepsis. Future studies should separate these groups given the known differences in host response.

Several other notable groups were also excluded: patients who required large amounts of oxygen support or ventilatory support, patients who could not consent to the study and patients who could not perform the necessary exhaled breaths into the study device. Due to this, our study cohort was skewed toward less acute septic patients. However, these are often the patients that are most challenging to identify among the many patients presenting daily to busy EDs.

As study subjects were identified, if they had blood cultures ordered, they could not be enrolled until after rooming and provider evaluation. This was a limiting factor in how quickly after ED arrival the patients could be enrolled. As per the inclusion criteria, patients were enrolled within 6 hours of ED arrival and this six-hour period usually included a number of hours waiting in triage. As the majority of workup orders (labs and imaging) are placed at the same time, we do not expect that there would be a significant amount of care (IV fluids, antipyretics, anti-inflammatories, antibiotics, etc.) to be provided between the time of orders and the time of enrollment.

The analyzer used in the study was acceptable for this feasibility study but would not be practical for routine clinical use. It was expensive ($60,000), heavy (over 20 kg), and required continuous AC power. Meanwhile, patient compliance with the exhale rate expectations was difficult.

This study was conducted at two large tertiary care hospitals. One of the hospitals is in the center of a large city and provides care to a considerable indigent population. As such, the study results may not be generalizable to other hospitals with different patient populations.

Finally, an unknown which needs further description is the biphasic nature of NO in sepsis; the duration of time a septic patient has elevated NO levels before evolving to abnormally low levels is unknown. However, despite this unknown, the VSNO score was still able to increase the sensitivity of the detection of sepsis two-fold from the baseline in our data. Future studies could track serial NO levels through the infection and sepsis disease states.

## 6. Conclusions

Nitric oxide as part of a clinical score including triage vital signs demonstrates a high sensitivity for detection of bacterial infection and sepsis in ED patients presenting with concern for infection.

## Figures and Tables

**Table 1:** Calculation of VSNO score with comparison to SIRS score.

SIRS score	VSNO score
1 Pt: body temperature > 38°C or < 36°C	1 Pt: body temperature > 38°C or < 36°C
1 Pt: HR > 90 beats per minute	1 Pt: HR > 90 beats per minute
1 Pt: RR > 20 breaths per minute	1 Pt: RR > 20 breaths per minute
1 Pt: WBC > 12,000/mm^3^ or < 4000/mm^3^	1 Pt: nitric oxide (flow corrected ppb) < 7 ppb or > 12 ppb

**Table 2:** Patient characteristics.

	Overall	No sepsis	Sepsis
Total	104	50	54
Age, M ± SD	56.7 ± 18.1	57.5 ± 18.6	55.9 ± 17.8
Female, *N* (%)	41 (39.4)	23 (46.0)	18 (33.3)
Smoker, *N* (%)	13 (12.5)	7 (14.0)	6 (11.1)
Temperature, initial, M ± SD	37.4 ± 1.0	37.0 ± 0.9	37.7 ± 1.0
HR, initial, M ± SD	102 ± 18	94 ± 19	110 ± 14
SBP, initial, M ± SD	129 ± 23	128 ± 22	129 ± 25
DBP, initial, M ± SD	79 ± 16	79 ± 16	80 ± 17
RR, initial, M ± SD	18.7 ± 3.2	17.7 ± 3.3	19.6 ± 2.9
Creatinine, initial, median (IQR)	0.96 (0.79–1.29)	0.96 (0.78–1.12)	0.98 (0.82–1.42)
Platelets, initial, median (IQR)	216 (167–281)	214 (161–276)	224 (167–299)
Bilirubin, initial, median (IQR)	0.70 (0.40–1.60)	0.70 (0.40–1.90)	0.70 (0.50–1.30)
Bacterial infection, *N* (%)	62 (59.6)	15 (30.0)	47 (87.0)
Viral infection, *N* (%)	7 (6.7)	0 (0.0)	7 (13.0)
Hospitalization, *N* (%)	87 (83.7)	38 (76.0)	49 (90.7)
Hospital total days, length of stay, median (IQR)	3 (2–6)	2 (1–5)	4 (3–7)
Any vasopressor need, *N* (%)	2 (1.9)	1 (2.0)	1 (1.9)
ICU stay, *N* (%)	3 (2.9)	2 (4.0)	1 (1.9)
In-hospital death, *N* (%)	0 (0.0)	0 (0.0)	0 (0.0)

**Table 3:** Test characteristics and score characteristics.

	Overall	No sepsis	Sepsis
Total	104	50	54
NO, median (IQR)	9.8 (5.6–17.0)	9.6 (3.9–15.2)	10.6 (6.0–17.1)
WBC, median (IQR)	10.7 (7.7–15.4)	9.1 (5.7–13.3)	12.2 (9.5–16.9)
SIRS using triage vitals (2+), *N* (%)	60 (57.7)	15 (30.0)	45 (83.3)
VSNO score using triage vitals (2+), *N* (%)	74 (71.2)	26 (52.0)	48 (88.9)
Procalcitonin, initial, median (IQR)	0.20 (0.09–0.78)	0.12 (0.07–0.22)	0.50 (0.15–5.97)
C-Reactive protein, initial, median (IQR)	8.4 (4.6–17.8)	6.1 (2.5–17.6)	10.1 (5.9–18.5)

**Table 4:** VSNO score diagnostic performance for detecting sepsis.

Test characteristics	VSNO	NO level alone (< 7 or > 12 ppb)	SIRS score using triage vitals only (no WBC)
Sensitivity (95% CI)	0.89 (0.77–0.96)	0.81 (0.69–0.91)	0.54 (0.40–0.67)
Specificity (95% CI)	0.48 (0.34–0.63)	0.32 (0.20–0.47)	0.80 (0.66–0.90)
PPV (95% CI)	0.65 (0.53–0.76)	0.56 (0.45–0.68)	0.74 (0.58–0.87)
NPV (95% CI)	0.80 (0.61–0.92)	0.62 (0.41–0.80)	0.62 (0.49–0.73)
AUC (95% CI)	0.68 (0.60–0.77)	0.57 (0.48–0.65)	0.67 (0.58–0.76)

**Table 5:** VSNO score's diagnostic performance for detecting bacterial infection.

Test characteristic	VSNO	NO level alone (< 7 or > 12 ppb)	SIRS score using triage vitals only (no WBC)
Sensitivity (95% CI)	0.82 (0.70–0.91)	0.82 (0.70–0.91)	0.40 (0.28–0.54)
Specificity (95% CI)	0.45 (0.30–0.61)	0.36 (0.22–0.52)	0.67 (0.50–0.80)
PPV (95% CI)	0.69 (0.57–0.79)	0.65 (0.54–0.76)	0.64 (0.47–0.79)
NPV (95% CI)	0.63 (0.44–0.80)	0.58 (0.37–0.77)	0.43 (0.31–0.56)
AUC (95% CI)	0.64 (0.55–0.73)	0.59 (0.50–0.68)	0.53 (0.44–0.63)

## Data Availability

The data that support the findings of this study are available from the corresponding author upon reasonable request.

## Supporting Information

Appendix 1: This is a list of all data variables that were collected during the study including variables from the EMR and clinical variables.
