# Supplementary material for: Evaluation of the Diagnostic Accuracy of Exhaled Nitric Oxide as a Marker of Infection and Sepsis in Emergency Department Patients
Source: Emerg Med Int. 2025 Mar 11;2025:8911242. doi: 10.1155/emmi/8911242 (PMC11986937; doi:10.1155/emmi/8911242)
Supplement: Supporting Information — Additional supporting information can be found online in the Supporting Information section. [file 8911242.f1.docx]

Appendix 1**:** Data collection details

| **Data Point** | **Collection Details** |
| --- | --- |
| *Clinical Data Points within 6 hours of FeNO testing* | |
| Patient Age | From chart |
| Patient Sex | From patient demographic chart data |
| Patient Weight | From chart |
| Patient Race & Ethnicity | From patient demographic chart data |
| Temperature | Highest reading |
| Heart Rate | Highest recording |
| Respiratory Rate | Highest recording |
| Oxygen Saturation | Initial value on presentation to ED; lowest value within 6 hours of FeNO testing |
| Oxygen Delivery Method | Maximum O2 delivery method within 6 hours of FeNO testing (Room Air, nasal cannula, facemask, BiPAP, intubation) |
| Systolic BP | Initial and Lowest recorded SBP |
| Diastolic BP | Initial and Lowest recorded DBP |
| Mean Arterial Pressure | Lowest recorded |
| Vasopressors | Yes or No; if yes, list of vasopressors |
| GCS | Lowest documented score; note that number value may be inferred from chart notes, i.e. “patient confused.” |
| Fluid Resuscitation | Milliliters of fluids administered while patient in the ED, inclusive of both crystalloids and colloids/blood products |
| *Laboratory Data Points within 6 hours of FeNO testing* | |
| Lactate | Initial value |
| Cultures | Yes or No; if yes, source location (i.e. blood urine, stool, sputum, swab, aspirate) |
| Culture Results | Growth/No Growth. If Growth:   - Blood cultures: Bacteria - Urine: Bacteria, and how many - Stool: Bacteria/virus - Sputum/Swab/Aspirate: Bacteria |
| White Blood Cells | Initial value |
| C-Reactive Protein | Initial value |
| Procalcitonin | Initial value |
| Creatinine | Initial value |
| Bicarbonate | Initial value |
| Viral Swabs | PCR Covid and Flu swab results |
| *Additional Data Points from Hospitalization* | |
| Imaging | Chest x-ray, ultrasounds, or CT/MRI results identify a source of infection? (Pneumonia, abscess, etc). Imaging data from within 24 hours of presentation |
| Source of Infection | Per clinician and other documentation in EPIC |
| Length of Stay | Length of stay in hospital and/or ICU |
| Admission to ICU | Time from ED presentation to ICU admission; total ICU LOS |
| 48-hour Return to ED | If discharged directly from ED, did the patient return to ED within 48 hours with a diagnosis of infection? - Yes or No |
| Mortality | In hospital mortality due to sepsis |
